# Treatment preferences of patients with relapsed and refractory multiple myeloma: a qualitative study

**DOI:** 10.1186/s12885-019-5467-x

**Published:** 2019-03-25

**Authors:** Janet A. Parsons, Nicole R. Greenspan, Natalie A. Baker, Chris McKillop, Lisa K. Hicks, Olivia Chan

**Affiliations:** 1 Applied Health Research Centre, Li Ka Shing Knowledge Institute, St. Michael’s Hospital, 30 Bond St., Toronto, ON M5B 1W8 Canada; 2 Department of Physical Therapy and the Rehabilitation Sciences Institute, University of Toronto, 160-500 University Ave., Toronto, ON M5G 1V7 Canada; 3 Toronto Public Health, 277 Victoria St., Toronto, Canada; 4 Dalla Lana School of Public Health, University of Toronto, 155 College St., Toronto, ON M5T 3M7 Canada; 5 Turalt, Inc., Suite 2201, 250 Yonge St., Toronto, Canada; 6 Division Hematology/Oncology St. Michael’s Hospital, Li Ka Shing Knowledge Institute, St. Michael’s Hospital, 30 Bond St., Toronto, Canada

**Keywords:** Haematological cancer, Treatment options, Survivorship, Side effects, Experiences of illness, Qualitative methods

## Abstract

**Background:**

Multiple myeloma is a haematological malignancy characterized by significant morbidity and mortality. This study sought to develop an in-depth understanding of patients’ lived experiences of relapsed or refractory multiple myeloma (RRMM) and its treatment, and to identify which features of treatment were most important to them.

**Methods:**

Qualitative interviews and focus groups (FGs) were conducted with 32 people living with RRMM across Canada. In Phase 1, interviews focused on participants’ accounts of their experiences with the disease and its treatment and laid the groundwork for the FGs (Phase 2). The FGs developed a deeper understanding of patients’ treatment priorities. Interview and FG transcripts were coded for emergent themes and patterns.

**Results:**

The interviews identified important side effects that had significant impacts on patients’ lives, including physical, cognitive, and psychological/emotional side effects. Participants also identified specific treatment features (attributes) that were important to them. These were compiled into a list and used in the FGs to understand patients’ priorities. Higher prioritized attributes were: life expectancy, physical and cognitive side effects, and financial impact. Mode of administration, treatment intervals, psychological side effects, and sleep/mood effects were identified as lower priorities.

**Conclusions:**

RRMM and its treatments impact importantly on patients’ quality-of-life across a range of domains. Patients prioritized treatment features that could enhance life expectancy, minimize side effects and offset financial burdens.

**Implications for cancer survivors:**

A clear articulation of patient priorities can contribute to efforts to design treatment with patients’ concerns in mind, thereby promoting a more patient-centered approach to care.

## Background

Multiple myeloma (MM) is the second most common haematologic malignancy, and is characterized by significant morbidity and mortality [1, 2]. There were an estimated 2700 new diagnoses of multiple myeloma in Canada in 2015 and it was responsible for 1400 deaths in the same year [3]. Typical age of onset is between 65 and 70 years, and with an aging population, its incidence is expected to rise [4, 5].Thanks to recent therapeutic advances, there have been overall improvements in survival times, with most patients living years with the disease [6–9]. Ultimately though, multiple myeloma is incurable and virtually all patients will relapse and/or become refractory to treatment, with the disease becoming more aggressive and drug resistant over time, with shorter response intervals [1]. Thus, the development of treatments that provide “durable disease control and symptomatic relief” for patients with relapsed or refractory multiple myeloma (RRMM) represents an unmet medical need [1, 10].

Most research to date has focused on clinical prognostic factors and therapies to target the complex pathogenesis of multiple myeloma [1, 6–8]. Relatively little attention has been paid to patients’ experiences of the disease in the published literature – still less so in the context of RRMM. Qualitative studies, using various methodologies, have outlined how living with uncertainty, symptom burden and quality of life concerns all impact on patients’ perceived body image, role function and social relations [4, 9, 11, 12]. A qualitative paper by Coon and Coleman (2007) reported on MM patients’ experiences of fatigue and their beliefs regarding exercise, but did not address lived experiences of the disease in a comprehensive way [13]. Another study focused on the ‘work of illness’ [14] engaged in by patients with MM and their caregivers, focusing on risk work (self-surveillance and risk evaluation) and emotional work necessitated by MM [15]. Even less is known about MM patients’ treatment preferences, and how these preferences are shaped by their long-term experiences of cancer survivorship. This is especially important given the significant quality-of-life issues faced by patients living with RRMM. One quantitative study reported on health-related quality-of-life (HRQL) in relation to treatment-free intervals [2]; however its use of quantitative survey methods did not permit an in-depth consideration of patients’ experiences or the contexts that influenced their experiences of care [11, 16]. In order to develop and deliver truly patient-centered care, the voices of patients must be brought into the priority-setting arena.

A common approach for studying patients’ treatment preferences is conjoint analysis (also known as ‘discrete choice experiment’ or DCE) [17–19] which has been used to elicit preferences of cancer patients [20]. The essential first step of DCEs is the collection of qualitative data, so that researchers develop a good understanding of which treatment attributes are prioritized by patients, which they present to them in the second quantitative step in a DCE [21]. The quantitative step involves administering surveys to a broader sample, to be ranked and analyzed [22]. While conjoint analysis has been conducted to assess treatment priorities of patients with multiple myeloma previously [23], unfortunately it has not focused on patients with RRMM specifically, who may have different priorities due to their longer-term experiences of being cancer patients.

Coast and Horrocks (2007) note that the qualitative research phases of conjoint analyses are typically under-reported, with little opportunity to assess the methodological rigour of this foundational step [21]. Our study fills an important gap in that it sought to understand the treatment preferences of people living with RRMM in a meaningful way, one that attended to the inherent complexities of their experiences of living with an incurable yet chronic disease characterized by an unpredictable and fluctuating course [4, 9, 15]. It was also designed to inform a future quantitative DCE study employing surveys. This paper reports the findings of this initial qualitative investigation.

### Purpose

Our purpose was to develop an in-depth understanding of patients’ lived experiences of RRMM and its treatment, and to identify which features of treatment (attributes) were most important to them. We had three specific objectives: 1) to elicit the perspectives of patients from across Canada regarding their experiences of RRMM and myeloma treatments; 2) to identify treatment attributes that patients viewed as important (e.g. mode of administration, effectiveness); and 3) to qualitatively rank treatment attributes in order to identify patients’ priorities.

## Methods

We adopted an approach of qualitative description in keeping with the pragmatic and descriptive purpose of our study [24] . Nevertheless, we were aware of sensitizing theoretical concepts that informed our approach to interpreting our findings, in particular those surrounding patient experiences of chronic illness [25] and particularly those characterizing cancer and its management as ‘work’ [14]. Theory can be brought to bear at any point in the research process [26], and we used theoretical contributions from the literature on *biographical disruption* [27] as a device for deepening and contextualizing our interpretation as outlined in the discussion section.

Our qualitative study had two data collection phases. The first phase entailed in-depth individual interviews with patients with RRMM to document their lived experiences of the disease and its treatment, and to describe treatment features (attributes) that they saw as important. The second phase consisted of focus groups, which concentrated on identifying patients’ *priorities* related to the identified treatment attributes. We sought to capture the perspectives of patients with RRMM from across Canada, which posed challenges due to Canada’s size and geographic variation. Participants were recruited using both purposive and convenience sampling methods [28] in order to maximize the variation of the sample (e.g. a mixture of men and women, varied age at disease onset, and from different regions of Canada). Recruitment was carried out with the assistance of Myeloma Canada, a national patient organization, which sent out study information and recruitment materials to its members. Interview participants were recruited from across Canada and participated in in-depth semi-structured one-on-one interviews by phone; follow-up focus groups were conducted in regions (Alberta, Ontario and Quebec) where interview participants had indicated they would be able to attend. Participants were screened for eligibility prior to participating in a phone interview or focus group. Eligibility criteria included having been diagnosed with relapsed and/or refractory multiple myeloma, having had 2 or more relapses, and being treatment experienced. Being treatment experienced was defined as having been treated with: 1) either bortezomib (VELCADE®) or carfilzomib (or both) AND 2) either lenalidomide (REVLIMID®), pomalidomide (POMALYST®), or Thalidomide (THALOMID®) (or any combination of these 3). All participants gave written informed consent and the study was reviewed and approved by the St. Michael’s Hospital Research Ethics Board (REB# 15–210).

### Data collection procedures

In Phase 1, the semi-structured interviews were conducted by telephone. The interview guide was designed to capture participants’ experiences of living with multiple myeloma and related issues. Topics covered included: treatments they had received and how these impacted their lives; experiences with their health care team and support systems; and financial impacts of their treatments. Participants were also asked questions about their preferences for certain features of treatments (e.g. importance of treatment-free periods, mode of administration, etc.). In-person focus groups were held in three cities in three Canadian provinces (Alberta, Ontario and Quebec). Each focus group lasted approximately 90 min.

Based on the results of our interviews, we derived a list of features that we presented to focus group participants. Employing an adapted nominal group technique [29], participants were asked to reflect on the list and add any treatment features that they perceived to be missing. This was discussed as a group. They were then asked to rank the list of features in order of importance to them on paper, which they completed individually. These individual rankings were averaged by the facilitators during the focus group, and the new aggregate list of prioritized treatment features were presented to and discussed as a group. Based on these discussions, changes were made to the list to reflect participants’ perspectives. Interviews and focus groups were audio recorded, transcribed by professional transcriptionists, and checked for accuracy [28].

### Analysis

Four experienced qualitative researchers (NRG, CM, NAB, JAP) conducted the data analyses, with input from a fifth team member who assisted with running the focus groups (OC). Interview data were analyzed first (CM, NAB, JAP) to identify common themes and treatment attributes, which then informed the analysis carried out on the focus group data (NRG,OC, JAP). An iterative and inductive approach was employed, combining features primarily of qualitative description [24] supplemented with those of grounded theory [30], whereby the coding and themes are developed using the language and descriptions elicited from participants. During both phases, analysis entailed reading transcripts multiple times, and coding the transcripts for emerging themes and patterns [31–34]. A constant comparison approach was used to ensure that codes and content were compared throughout the data corpus [35]. NVivo 11 software facilitated data management. Techniques for promoting analytic rigour included the use of multiple analysts, multiple data sources, as well as checking and questioning [32, 36]. While saturation of themes was achieved from the interviews (meaning that little new information was gained by subsequent interviews) [37], this was based on the modest goals of the study, which were to develop a list of treatment attributes important to RRMM patients that were meaningful to them, and which could be used in the focus groups as well as informing future DCE studies with more participants.

## Results

### Participants

Overall, 32 participants from across Canada were involved in this study: 22 men and 10 women (see Table 1). In Canada, multiple myeloma is slightly more common in men than women [3].

**Table 1:** Participant characteristics (*N* = 32)

Sex	22 Men 10 Women
Age	Range: 51–83 yrs. old Average: 66
Marital Status	29 Married or Common-law 3 Divorced
Province	5 British Columbia 11 Alberta 2 Manitoba 9 Ontario 4 Quebec 1 Newfoundland
Year Diagnosed	Range: 1991–2015 10 Diagnosed in 2005 or before

Twenty-three people participated in interviews, and 16 participated in focus groups. 7 participants who were interviewed also attended one of the focus groups. Two focus groups consisted of 10 and 4 participants respectively, while one was much smaller and consisted of only 2 participants. While more participants were planned for this third group, some participants who originally planned to attend were unable to do so due to ill health.

### Themes from interviews

During the interviews, the study participants offered in-depth accounts of their experiences of multiple myeloma and the treatments they had received. Myeloma and its treatment were portrayed as affecting many aspects of their lives: from the physical toll exacted by disease progression to the side effects of treatment that had physical, emotional/psychological dimensions. While treatments that prolonged life were appreciated, they recounted in detail how the side effects of treatment also resulted in profound disruption to their daily lives.

#### Side effects – physical

Participants described a myriad of physical effects that they experienced both as a result of their disease and that they attributed to the treatments they received. Fatigue, musculoskeletal pain, gastrointestinal symptoms, neuropathy, and neutropenia all featured in their accounts. Some participants commented on sleep and other mood disturbances that they linked to steroid therapy (specifically dexamethasone). All of these experiences of physical effects were seen to have important impacts on their social relations (including social and role functioning), their self-image, and their emotional well-being.

Virtually every participant indicated that **fatigue** was an important concern for them. This was described as not just being ‘tired’, but exhausted to a degree that it impacted many aspects of their lives. They said it was hard to concentrate on tasks, to go to work, or to undertake their usual activities of daily living. As a result it had a major impact on their lives.

*“I think the tiredness, I mean I go to bed now usually around 9:00 and I think that’s ridiculous…. I felt if I don’t get out of bed I will be in bed for the whole day, so I sort of forced myself out of bed and go downstairs, have breakfast, do this sort of stuff you know, a few normal things…”* (MM3)

Most participants spoke at length about various forms of musculoskeletal **pain**. This was primarily related to bone pain and many of the participants reported experiencing fractures. Fear of future fractures impacted other aspects of their lives, forcing some of them to give up valued sporting or leisure activities. One participant commented on breaking a rib simply by coughing. Participants also reported muscle cramping and soreness.

**Gastrointestinal (GI) symptoms** also featured prominently, ranging from diarrhea to constipation to nausea and vomiting. This in turn had effects on appetite and weight loss and some participants said they spent considerable time managing their diets in an attempt to control these unwanted effects. GI symptoms were not characterized as minor, and could last years in some instances. Participants also told how GI symptoms impacted their ability to leave the house, to plan other valued activities, including social activities.

*“The worst side effects were when I was getting chemotherapy … I would have to watch my diet so strictly or else I would end up with a weekend where I was sitting on the can or else throwing up.”* (MM19)

Quite a few participants recounted that **neuropathy** impacted their overall function. Not being able to feel one’s feet was characterized as unsettling, with important implications for mobility. Other examples participants attributed to neuropathy included trouble with essential functioning, from eating to going to the bathroom. This was further discussed as impacting participants’ basic quality of life and a few participants even talked about how this would diminish their desire to continue living.

Participants often used the term ‘**neutropenia**’ outright, reflecting the expertise with medical terminology they had acquired from living with the disease. They engaged in significant monitoring of their blood counts, and knew which levels were acceptable and which were worrisome. The associated risk of infection impacted their quality of life directly. Participants said they were instructed to avoid crowds and to avoid those with colds and other infections. This meant that they sometimes felt isolated, even from members of their own family. One participant commented that when her counts were particularly low she was not able to see her grandchildren.

Physical effects could also include sleep disturbances as a result of **steroid therapy**. As one participant commented about the side effects of dexamethasone (‘dex’),

*“The problem is with dex you don’t sleep. So you have all the sleep deprivation issues that come along with it. I don’t know how to make it any clearer, there are no free rides. I don’t know anybody that’s dealing with myeloma that doesn’t have drug issues.”* (MM27)

#### Side effects - cognitive

A number of participants referred to the cognitive effects that they attributed to treatments. One participant commented, *“I am losing my train of thought all the time, and that’s one of my side effects of my chemo that I blame it on”* (MM20). Another participant stressed:

*“It affected my cognition, my retrieval of information…There has been some improvement since I stopped everything but not really that much. I think probably there is some permanent damage done.”* (MM12)

Difficulty concentrating and memory lapses were characterized as disturbing by participants. Descriptions of cognitive effects were often overlapping with other effects – of treatment and ongoing, relentless disease management. Fatigue, emotional lability (highs and lows –see below), pain, sleep disturbances, and the effects of steroid medication were all portrayed as linking to this experience of ‘cog fog’ that is alluded to in other cancer populations [38].

#### Side effects – Emotional and psychological

As noted above, some participants attributed some of their (frequently overlapping) physical and psychological effects to the steroid dexamethasone. One participant characterized it as *‘demon dex or devil dex’* to capture his feelings about it. Participants described intense highs and lows (mood swings), periods of feeling intensely energized followed by bouts of extreme fatigue. A participant commented,

*“…on dex days I am flying, but on the dex days now I basically get out of bed and basically do screw all until I go back to bed.”* (MM27)

*“The worst drug[s] we take are the high dose – well the steroid, the dexamethasone, because that does impact one’s personality, and it can have a lot of collateral damage with caregivers and people around you…also, in my case because I knew what the side effects were, the mania and just hyper-energetic and just intolerable to be with, I basically withdrew socially.”* (MM15)

Participants spoke at length about the psychological burden (and consequences) of living with multiple myeloma and the effects of treatment, and the interviews themselves were sometimes emotional. Managing the psychosocial effects of living with a chronic (but ultimately fatal) medical condition was portrayed as further ‘work’ that they did.

*“I think that having something to focus on, it wouldn’t matter what that was I don’t think, but you need to focus on something and you need to keep your mind occupied. Because I am sure you could get incredibly, incredibly depressed, it would be so easy you know. But who’s that going to help?”* (MM21)

These effects had important implications for their broader quality of life, often disrupting their social relations and their ability to work and pursue valued activities.

Finally, interview participants also commented on what ideal treatments might look like, which included specific features of treatments, such as: modes of administration, improved life expectancy, treatment intervals, relation to other treatments, physical side effects, sleep and mood side effects, cognitive side effects, psychological/emotional side effects, financial impact and limited steroid use.

### Priority of treatment attributes

Based on our preliminary analysis from the interviews, we compiled a list of 10 features of treatment that were important to participants, which were used to discuss priorities with patients during the focus groups; a further two features were added by focus group participants (see Table 2) [29]. All of the features were discussed in depth and ranked during the focus groups, and the ranked results from each group were compared to one another to come up with the priorities discussed below.

**Table 2:** List of Treatment Attributes

Feature	Description
Higher priority	
Life expectancy	The amount that a treatment prolongs life (or not)
Physical side effects	Does not increase common side effects such as fatigue, digestive problems, nausea or neutropenia
Cognitive side effects (‘Chemo brain’)	Does not contribute to memory or concentration problems
Financial impact	How the costs of treatment are covered (e.g. by government, insurance etc.)
Mixed priority	
Limited steroid use	Drug treatment does not require dexamethasone (‘dex’) or very low doses of dex are administered
Relation to other treatments	Does not preclude other treatments or make other treatments ineffective
Lower priority	
Mode of administration	How treatment is given, for example orally (taken at home), or IV treatment at the hospital
Treatment intervals	Allows for “down-time” or “off-treatment” breaks during treatment periods
Psychological/emotional side effects	Treatment side effects are predictable so that you are able to make plans.
Sleep and mood side effects	Does not increase sleep disturbances and mood swings
Lowest priority	
^a^Accessibility	The treatment and the healthcare provider are accessibly located
^a^Distribution	Smooth and consistent access to medications (financial and logistic)

^a^These treatment features were added by focus group participants

The focus groups shared several higher, lower and lowest priorities in common. There were also two features of treatment that the focus groups did not agree on, where one focus group considered it a higher priority, and another considered it a lower priority.

#### Higher priority treatment features

The higher priorities were life expectancy, physical side effects, cognitive side effects, and financial impact. Life expectancy – the amount of time that a treatment prolongs life – was clearly a feature of treatment that was important and many participants talked about its importance. For some, it was clearly a higher priority than everything else.

*“The way I kind of look at it was if it works well, and side effects, I don’t really care too much about, I mean to a certain extent obviously, but if it’s working well I can deal with the side effects…. Life expectancy [is] #1”* (MM31)

However, for others, the physical and cognitive side effects of treatment were characterized as just as much a priority as life expectancy. Their quality of life, which they said was reduced because of side effects, was considered just as important as longevity. Several participants discussed how their priorities had changed over time, and how being a long term survivor with RRMM had changed their perspectives. One participant explained how this affected his priorities about life expectancy and quality of life:

“*When I was diagnosed my [children were in grade school] and I was [under 40 years] old, so life expectancy was #1... whereas 25 years later life expectancy is not that great, [physical and cognitive side effects, and the effects of dex] are more important… Quality of life is more important.”* (MM5)

While a number of side effects were discussed by participants, physical and cognitive side effects were higher priorities in terms of maintaining a good quality of life.

*“So, like in the end you know, we were, we all recognize that there is no cure so we look for a treatment that gives us the best quality of life and in that you know, we want to still have a good physical capacity and good mental capacity as best we can, right? That’s what we strive for and as we live longer with the disease, we strive for even more of that.”* (MM17)

These accounts suggest that patients’ priorities may change over time and may be dependent on where in their RRMM journey they find themselves. This speaks to priority-setting as a complex, nuanced and fluid process.

Financial impact was also given a higher priority by focus group participants. Financial impacts could be stressful, and could represent a major burden on people without sufficient resources, including those with younger families.

*“The stress... I mean the financial impact... is really stressful. [And now that there are younger people being diagnosed as] young as... mid 40s... this even plays a bigger role... Because they have to stop their work and some are trying to go to work and because of the stress of the financial impacting young children and all of that”* (MM33).

#### Mixed priority treatment features

There were two instances where features were considered a higher priority in one focus group, yet another group considered it a lower priority: limited steroid use and relation to other treatments.

Limited steroid use was a feature of treatment that was ranked as a top priority by the focus group that had a lot of long term survivors. However, even in the focus group that did not rate it as a higher priority, there was a lot of discussion about the particular effects of steroid use. Many participants described how the impacts of steroid treatment challenged numerous aspects of their everyday lives.

*“Dex is different for everybody... [But for me] because there’s so many side effects it’s just, it really changes my life basically.”* (MM11)

The specific effects participants described were diverse, but a few mentioned that the severity of the effects that they experienced, and their impact on others around them were a significant challenge. In some of the discussions, it became clear that some participants saw limited steroid use and cognitive side effects as the same thing – or at least very closely intertwined. This may explain why one group did not include limited steroid use as a higher priority, because they did include cognitive side effects as a higher priority.

There was also some confusion surrounding relation to other treatments; some participants confused this with the situation when multiple myeloma treatments precluded treatment for other illnesses. In addition, confusion about relation to other treatments could also be about whether they had a feeling of agency in their treatment options, or if they felt their doctor had all the power in decision-making. This suggests that patients’ understanding and prioritizing of a treatment’s preclusion of other treatments may be interrelated with whether they felt they were part of the treatment decision-making process or not. This highlights the complex relationship between patients, their healthcare providers, and available treatments. There may be situations where ‘treatment options’ are not perceived to be ‘options’ at all by patients and this may depend on the particular healthcare providers and/or the patients themselves.

#### Lower and lowest priority treatment features

The lower priorities were: mode of administration, treatment intervals, psychological side effects and sleep and mood effects. While these features of treatment were discussed, and some participants had particular concerns related to them, overall, they prioritized them lower than others.

The lowest prioritized features of treatment – accessibility and distribution – were the two features that participants added in the focus groups (they were not mentioned in interviews). The accessibility of treatment, which referred to treatment and healthcare providers being accessibly located, was discussed by a focus group participant who was thinking about retiring and moving provinces, and seemed important to some individuals, but it was not, overall, seen as a priority. However, focus groups did discuss their questions and concerns about what drug coverage was like in other provinces, and hoped that the treatments they needed were accessible. The distribution of medications, where the access to them was financially and logistically smooth and consistent was mentioned as a feature of treatment by one participant, but overall, not seen as a priority.

#### Overall aim: a ‘normal’ life

In our study, participants discussed how their disease and treatment effects impacted many aspects of their quality of life. They discussed the profound impact their disease had on their lives, the profound disruption they experienced and how they had to adapt to a “new normal” since diagnosis.

*“It’s 100% impact. You know you can’t – you hear all the tired clichés you know, you got to do this, fight the battle, it’s all bullshit. You know the bottom line is that this disease takes over your whole life. I don’t care what cancer it is, I don’t care who you are, it takes over your whole life. So from the day I was diagnosed until today, everything is different. So now it is the ‘new normal’.”* (MM27)

Participants commented that there were multiple ways in which they had to adapt to ‘living with uncertainty’. This could be in terms of when and how they might relapse, how much longer they had left to live, or even the predictability of certain treatment effects. Participants were not expecting a ‘cure’ or a remission that would last ‘forever’ (great though that would be), but the prospect of relapse was portrayed by some as an ongoing presence in their lives. They expressed gratitude for times when they could ‘normalize’ their lives and not attend to the work of managing their disease. Overall, what they wanted most from their treatments was assistance in maintaining as ‘normal’ a life as possible.

*“Being a myeloma patient… with these… remissions, refractory relapses… it’s like a roller coaster. I mean it’s an emotional roller coaster, it’s a physical roller coaster and… if we could just even out that a little bit”* (MM33)

*“[Overall, I hope my treatments for RRMM will help me] to feel good, to have energy, to be able to function, to be able to do things, have what they call a normal life...”* (MM30)

Overall, participants reflected on how the disease and its treatment affected many aspects of their lives and that these were intricately woven together. RRMM impacted profoundly on their emotional well-being and their social relationships, especially their families, and had implications for their identity/self-concept, their views of the future, and how they defined a ‘normal’ life. What they wanted most from their treatments was assistance in maintaining as normal (and pleasurable) a life as possible.

## Discussion

This study focused on the patient journeys of those who have experienced RRMM and offers a valuable contribution to the literature. It offers an in-depth understanding of patients’ lived experiences of RRMM, the effects RRMM and its treatment has on their lives, as well as identifying which effects are most important to them. It also documents attributes of treatments for this disease which are important to patients, and identifies which features/attributes they prioritize. There was a high level of congruence among participants surrounding treatment priorities. Quality of life issues, such as a lack of physical and cognitive side effects, become as important as prolonging life for patients who are long-term survivors with RRMM.

These findings bear similarities to those from other studies, especially as they relate to cancer patients’ experiences of quality of life issues that physical symptoms such as fatigue introduce [9, 11, 12, 39]. For example, the difficulties our participants recounted in managing the side effects of medication echoes findings from other studies [9]. However, our findings also differ in important ways. For instance, our study differs from that of Mulbacher’s (2008) quantitative assessment of treatment preferences: in both studies, prolongation of life expectancy was prioritized as a treatment feature. However, Mulbacher’s (2008) participants prioritized further treatment options, while our participants did not. Also, our participants’ prioritized financial impacts, while theirs did not. This may reflect differences in the delivery and funding of health care in the German versus Canadian contexts. Our study also begins to fill gaps identified by Osborne and colleagues (2014), who noted that existing (quantitative) QOL measures, including those used in MM, do not capture all the issues important to MM patients [16]. Our study serves to fill in some of this missing information, including how patients prioritize treatment attributes.

Beyond the original purpose of the study, which was to catalogue the treatment attributes that patients with RRMM find meaningful, and to understand how they prioritize these attributes, our study’s findings link to the broader literature on patient experiences of cancer and its treatment. For the participants in our study, RRMM and its treatment represented a profound and ongoing *disruption* to their lives. This notion of *biographical disruption* has been applied to experiences of both acute or chronic illness, and has a longstanding history in qualitative health research [27, 40, 41]. It is a useful one for framing our study’s findings within a broader scholarly context. The nature of RRMM is that it affects primarily older individuals, at a time when they may be retiring or contemplating retirement in many instances. For many people in Canada, retirement is frequently viewed as a time when one has more time to enjoy family and friends and to fulfill long-deferred experiences, such as engaging in travel and leisure activities. Instead, our participants’ accounts could be interpreted as experiences of unrelenting illness ‘work’ [14, 42] necessitated by managing both the disease and its treatment effects. They recounted that many of the taken-for-granted activities of older adults (such as spending time with their grandchildren) were curtailed or prevented. Participants expressed hopes that they might live a “normal life” in future, whereby they would have the energy and ability to engage in the activities and roles that they value. This takes this dataset beyond the issue of what a specific treatment might offer in terms of limited ‘side effects’ and instead moves us into the realm of patients’ aspirations of what their lives might look like.

Like any research, there are limitations to our study. This is a qualitative study intended to capture a range of important perspectives, but it is not meant to be an exhaustive account and is not intended to be generalizable at the population level. Rather it generates important insights and concepts that may be transferrable to other contexts. Our sample was composed mostly of participants who were married or in common-law relationships; it is possible that persons living alone may experience different issues. While MM is only slightly more common in men than women, men were also overrepresented in our sample, which may have been a reflection of convenience sampling. And while it was extraordinarily valuable to have the support of a national patient organization (Myeloma Canada) to assist us with recruitment, there is always the possibility that this may have introduced an element of self-selection, in that our sample may have represented a group of patients who were especially knowledgeable about and engaged in the management of their myeloma. Another related issue was that our participants were experiencing *relapsed and/or refractory* MM, which meant that many of them were experiencing frailty. This made it difficult for many of them to attend focus groups. Fortunately, by using telephone interviews, perspectives of those who were too frail to attend the focus groups were captured. Other authors acknowledge frailty as an issue with respect to treatment tolerance [5] and we would argue that this relates to tolerance for research activities as well. Another limitation was that we did not track the temporal profile of specific ‘side effects’ (whether they were ‘short-term’ or ‘long-term’) nor did participants typically relate side effects to specific medications. Rather, the effects of disease, effects of treatment, and other effects were less differentiated in participants’ accounts. Our study was designed to focus on ‘treatment attributes’ more generally. Finally, our study was shaped by the geographical dispersion of people living with RRMM in Canada, which had implications for who could participate in in-person focus groups.

## Conclusion

This study has documented the treatment priorities and lived experiences of patients living with RRMM. As such it offers an important contribution to the literature, in that it addresses the complexity and interrelatedness of patients’ physical, social, emotional and financial well-being, and weaves these into issues of priority setting. It also lays essential groundwork for future priority-setting research using quantitative approaches such as DCE. Our study has important implications for cancer survivors: a clear articulation of their priorities can contribute to efforts to design treatment with patients’ concerns in mind, thereby promoting a more patient-centered approach to care.

## Abbreviations

DCE: Discrete choice experiment
FG: Focus group
GI: Gastrointestinal
HRQL: Health related quality of life
MM: Multiple myeloma
RRMM: Relapsed or refractory multiple myeloma
